# The optimal sex pheromone release rate for trapping the codling moth *Cydia pomonella* (Lepidoptera: Tortricidae) in the field

**DOI:** 10.1038/srep21081

**Published:** 2016-02-16

**Authors:** Wei Liu, Jing Xu, Runzhi Zhang

**Affiliations:** 1 CAS Key Laboratory of Zoological Systematics and Evolution, Institute of Zoology, Chinese Academy of Sciences, #1 Beichen West Rd., Chaoyang, Beijing 100101, China; 2 University of Chinese Academy of Sciences, Beijing 100049, P. R. China; 3 State Key Laboratory of Integrated Management of Pest Insects and Rodents, Beijing 100101, China

## Abstract

For successful pest management, codlemone (E, E-8,10-dodecadien-1-ol) is widely used to monitor codling moth. The pheromone release rate is essential for the lure’s attractiveness. The optimal sex pheromone release rate (V_0_) for trapping codling moth was evaluated during 2013–2014. The overwinter generation V_0_ was 6.7–33.4 μg wk^−1^, and moth catches (MCs) were 0.82 ± 0.11 adults/trap/week; MCs for lower (V_1_) and higher (V_2_) release rates were 52.4% and 46.3%, respectively, of that for V_0_. The first generation V_0_ was 18.4–29.6 μg wk^−1^, with MCs of 1.45 ± 0.29 adults/week/trap. V_1_ and V_2_ MCs were 34.5% and 31.7%, respectively, of those for V_0_. Combining across generations, the final V_0_ was 18.4–29.6 μg wk^−1^, with MCs of 1.07 ± 0.06 adults/week/trap. V_1_ and V_2_ MCs were 51.4% and 41.1%, respectively, of that for V_0_. Overwinter generation emergence was relatively concentrated, requiring a wider V_0_. Maintaining the release rate at 18.4–29.6 μg wk^−1^ could optimize the lure’s efficacy; this resulted in the capture of nearly 1.9 and 2.4 times more moths than V_1_ and V_2_, respectively. The results also indicate that a dispenser pheromone release rate of 200–300 times that of the female moth can perfectly outcompetes females in the field.

The codling moth, *Cydia pomonella* (L.) (Lepidoptera: Tortricidae), is an important pome fruit pest all over the world and causes serious damage to apple (*Malus domestica* Borkhausen), pear (*Pyrus communis* L.) and walnut (*Juglans regia* L.)^1^,^2^. It is not only an invasive species but also an important quarantine pest in China. To date, the codling moth has been found in 7 provinces in China, including Xinjing, Gansu, Ningxia, Inner Mongolia, Heilongjiang, Jilin and Liaoning, posing a grave threat to the two major apple production areas in China, Shandong and Shanxi provinces^3^.

Trapping the male codling moth using its sex pheromone (codlemone, E, E-8, 10-dodecadien-1-ol) is the most successful technique for the monitoring of this species. The initial loading rate is an important criterion for the manufacture of dispensers and has been widely studied. A commonly used loading rate for codling moth monitoring is 1 mg sex pheromone^4^,^5^,^6^. However, the same loading rate may have different release rates due to the use of different dispenser materials and/or under different environmental conditions, leading to different trapping efficiencies. Therefore, the loading rate is not a reasonable criterion to use to evaluate whether a sex pheromone dispenser performs well. Whatever dispenser is used, the amount of sex pheromone released to the surrounding air per unit time (sex pheromone release rates) is the key factor that determines adult catches. The pheromone release rate that achieves maximum moth catches is termed the optimal sex pheromone release rate (V_0_). V_0_ can catch significantly more adults than lower (V_1_) or higher (V_2_) release rates.

A previous study has reported the response of the codling moth to different release rates of codlemone in the field in its native distribution area, Spain. At a range of 11–1078 μg d^−1^ (77–7546 μg wk^−1^), the pheromone release rate of 11–67 μg d^−1^ (77–469 μg wk^−1^) achieved the maximum male catches^7^. However, as an invasive pest in China, the codling moth has experienced a long period of diffusion and dissemination, adapting to various ecological environments and diversified host plants, which leads to different V_0_ for its trapping. In addition, the previous study^7^ did not use pheromone release rates <77 μg wk^−1^ and did not consider studying different generations.

## Materials and Methods

### Field trial location

The field trial was conducted in Doning County, Mudanjiang City, Heilongjiang Province (P.R.C.) (N44°03′27.0″, E131°05′46.8″). In 2013, we conducted a trapping test in 1 orchard. The orchard’s area was 4.5 ha, and the cultivar was K9 apple. In 2014, we conducted trapping tests in 3 orchards. The orchard’s areas were 3.3 ha (orchard 1), 2.8 ha (orchard 2) and 4.0 ha (orchard 3). The cultivars were K9 apple (orchard 1); K9 apple, 123 apple, and apple-pear (a common planted pear variety in China which has apple-like shape) (orchard 2); and 5DN pear (orchard 3). The average tree age was 19–20 years in all of the selected orchards, and the plant spacing and row spacing were 3 m and 4 m, respectively. 1–2 spray of beta cypermethrin (1–2 sprays) was applied to each orchard. The orchards were nearly abandoned. The codling moth was first reported in this location in 2006 and had been colonizing the area for nearly 10 years.

### Sex pheromone dispenser and traps

We prepared the dispenser with different sex pheromone loading rates to acquire different sex pheromone release rates in the field. The dispenser (height: 1.4 cm, radius: 0.8 cm) was made of black, vulcanized polyisoprene rubber (Institute of Zoology CAS; Chinese patent: CN201217257). Before loading, the dispensers were immersed in 75% ethyl alcohol for 24 h to remove impurities. Codlemone (>97% purity) was used as the sex pheromone. It was provided by Bedoukian^®^ Research Inc. and was dissolved into a mixed hexane + dichloromethane (9 + 1 by volume) solvent. Sex pheromone solution (10 μl) was loaded in the dispenser and absorbed after approximately 3 h. The dispensers were wrapped in aluminum foil, packed in PE bags and stored in the refrigerator at 4 °C.

The sex pheromone solution was prepared at a concentration range from 0.00001 to 0.3 mg μl^−1^ in 2 experiment years. In 2013, dispensers with 15 different loading rates (0.0001–5 mg, see Appendix Table 1) were prepared according to the method mentioned above, except that the 5 mg dispenser was loaded with 0.1 mg μl^−1^ codlemone solution 5 times at 20 min intervals. In 2014, dispensers with 9 different loading rates (0.05–10 mg, see Appendix Table 2) were prepared according to the method mentioned above with the exception of the 5 mg and 10 mg dispensers. These two sex pheromone dispensers used a larger dispenser (height: 3.4 cm, radius: 0.8 cm). In all, 50 μl and 100 μl sex pheromone at a concentration of 0.1 mg μl^−1^ were loaded in the 5 mg and 10 mg dispensers, respectively. All of the dispensers with the various loading rates were hung in a well ventilated room (April-June: temperature, 21.4 °C; relative humidity, 48.0%; wind speed, 2.3 m/s) for 0, 2, 4, 8 or 12 weeks before the trapping test. The combination of loading rates and pre-hang times could easily generate more sex pheromone release rate data than the loading rates alone. Each combination had a corresponding pheromone release rate. For example, a 1 mg loading rate corresponds to 1 pheromone release rate data point, but 1 mg × 0 weeks and 1 mg × 4 weeks correspond to 2 pheromone release rate datapoints.

The dispensers were placed in delta traps. The delta traps were provided by Pherobio Technology Co. Ltd. The trap was made of white plastic board; its cross section was an isosceles triangle with bottom side length: 13.5 cm, side length: 12.5 cm; the longitudinal length was 25 cm. Two holes were drilled into the side of the board to facilitate placement of the dispenser. A sticky board (13.5 × 25 cm^2^) was set in the trap to capture the moths.

### Field trapping test

Two experiments were conducted in 2013. Each experiment lasted a week (6.8–6.14; 7.30–8.5). The first experiment was conducted for the overwinter generation, and 11 dispensers with different loading rates were tested. The sex pheromone release rate range was 0.050–1169.0 μg wk^−1^ (Appendix Table 1). The second experiment was conducted for the first generation. Seventeen dispensers with different loading rates were tested (7 more than for the overwinter generation), and the release rate range was 0.017–1680.0 μg wk^−1^ (Appendix Table 1). The catch data for the 0.5 mg and 1 mg sex pheromone dispensers were used to correct the population variance of the various experiments. Five replicates of each trap with a different sex pheromone release rate were set. In total, 55 traps were placed in the orchard in the first experiment and 85 traps in the orchards in the second experiment. The traps were suspended at a 1.5–1.8 m height, with 5 trees and 4 rows between traps. The distance between each pair of traps was 18–20 m. The outer edges of the trapping zone were more than 10 m from the border of the orchard. All traps were set in the orchard using a completely randomized design. Their positions were reset using the same method on the third and fifth day of the experiment, and the trapping data were recorded then.

Four experiments were conducted in 2014 (5.24–6.6 and 7.22–8.4). The first period coincided with the codling moth overwinter generation (experiment 1–2) and the second with the first generation (experiment 3–4). For 5.24–5.30 (the overwinter generation), the dispensers with 9 different loading rates (0.05–10 mg) that had not been pre-hung were set, and their release rates were 8.0–681.5 μg wk^−1^. In 2014.5.31–6.6 (the overwinter generation), dispensers with 9 different loading rates that had been pre-hung for 4 weeks were set (0.05–10 mg), and their release rates were 0.5–1797.9 μg wk^−1^. In 2014.7.22–7.28 (the first generation), dispensers with 9 different loading rates (0.05–10 mg) that had not been pre-hung were set, and their release rates were 6.9–965.4 μg wk^−1^. In 2014.7.29–8.4 (the first generation), dispensers with 2 loading rates (0.2 mg and 1 mg) were set; the 0.2 mg loading rate dispensers had been pre-hung for 0, 2, 4, 8, and 12 weeks, and the 1 mg loading rate dispensers had been pre-hung for 0, 2, 4, 8, and 12 weeks. The release rates of those dispensers were 15.6–280.5 μg wk^−1^ (Appendix Table 2). Each experiment lasted 1 week. Extra 0.5 mg and 1 mg sex pheromone dispensers were set to correct the population variance of the different experiment times and blocks. Five replicates of each trap with a different sex pheromone release rate were set. In experiments 1–3, 165 traps were set in each trapping test. In experiment 4, 180 traps were set. The traps were suspended at a 1.5–1.8 m height, with 5 trees and 4 rows between traps. The distance between each pair of traps was 18–20 m. The outer edges of the trapping zone were more than 10 m from the border of the orchard. All traps were set in the orchard using a randomized block design. Each orchard was divided into 5 blocks based on the trap replicates. Each block contained traps with all of the release rates used in each experiment. Their positions were reset using the same method on the third and fifth day of the experiment, and the trapping data were recorded then.

### Sex pheromone release rates

All of the sex pheromone release rates in 2013 were estimated based on the actual test data obtained in 2014 (see Appendix Table 2). In ideal situation, the proportions of the sex pheromone amount released from the different loading rates dispensers were similar in certain time. Therefore the sex pheromone release rates of different dispensers could be estimated by proportions mentioned above × initial loading rates. First, the average proportion of pheromone release amount (AM) was calculated by dividing the pheromone release rate by the initial amount of pheromone. The AMs of the 4 experiments were as follows: 10.8 ± 1.4%, 15.5 ± 2.7%, 17.8 ± 1.1%, and 22.7 ± 2.1%. These values indicated that in our experiment, the dispensers with different loading rates could release, on average, approximately 10.8–22.7% of the initial amount of sex pheromone loaded during the 1 week trapping test. Therefore, the pheromone release rates in 2013 could be calculated by each dispenser’s pheromone loading rate × the AM of the 4 experiments. Then, the average value of the 4 release rates was considered the release rate of a given loading rate. That was in order to eliminate the effects of the environmental situation from different times, especially temperature. The 17 estimated sex pheromone release rates in 2013 were between 0.017–1670.0 μg wk^−1^ (Appendix Table 1) (release rates less than 0.1 μg/week were rounded to 3 decimal places).

In 2014, 10 dispensers were prepared for each combination of initial loading rate and pre-hanging time. Before trapping, 5 of them were shredded. The 0.05–3 mg sex pheromone dispensers were extracted in 2 ml vials with 1 ml hexane for 24 h. The 5 mg and 10 mg sex pheromone dispensers were extracted in 4 ml vials with 3 ml hexane for 24 h. The other 5 dispensers were used for the trapping test. After 1 week of trapping, the dispensers were retrieved and extracted using the same method just described. The average efficiency of sex pheromone extraction for the different loading rates was 50.4 ± 1.8% (Appendix Table 3) based on our method. The actual amount of sex pheromone in each dispenser was calculated by the dividing the gc-fid testing value by 50.4%. All extraction samples were stored at −20 °C.

A 500 μl solution was prepared for each extraction sample. Before testing, 100 μl n-heptadecane (Aladdin^®^, >99.5% GC, 1000 ng μl^−1^) was added to each sample as the internal standard. The samples were analyzed by gas chromatography with a flame ionization detector. The standard samples for the standard curve were prepared at the following concentrations: 1 ng μl^−1^, 10 ng μl^−1^, 50 ng μl^−1^, 100 ng μl^−1^, 1000 ng μl^−1^, and 5000 ng μl^−1^. Testing was performed by a gas chromatograph (Agilent 7890A; Agilent technology) equipped with an HP-5MS column with 5% Phenyl Methyl Solix (30 m × 250 μm × 0.25 μm). The oven was maintained at 50 °C for 1 min. The temperature was then increased to 100 °C at a rate of 10 °C/min and was then maintained for 8 min. After that the temperature was raised to 160 °C at a rate of 3 °C/min and then raised to 250 °C at a rate of 20 °C/min and maintained for 10 min. Each sample’s run time was 48.5 min. The injection volume was 0.2 μl.

The sex pheromone release rates were calculated by subtracting the sex pheromone amount after trapping from the sex pheromone amount before trapping. The sex pheromone release rate range was 0.5–1797.9 μg wk^−1^ (Appendix Table 2).

### Statistical analysis

**Figure 1:**
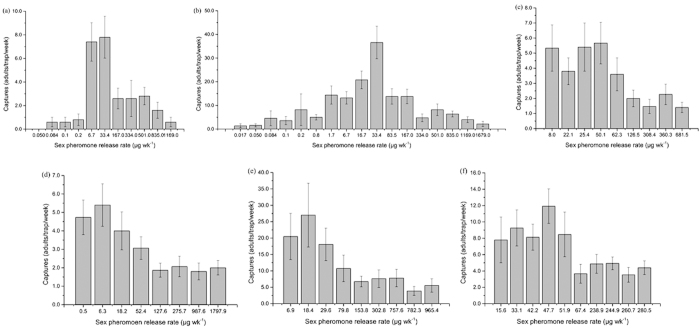
The original captured moth data of different sex pheromone release rate in 2013–2014 (the column was mean ± SE. (**a**) Over winter generation in 2013: 6.8–6.14, n = 5; (**b**) First generation in 2013: 7.30–8.5, n = 5; (**c,d**) Overwinter generation in 2014: 5.24–5.30, 5.31–6.6, n = 15; (**e,f**) First generation in 2014: 7.22–7.28, 7.29–8.4, n = 15).

The original trapping data of the traps arranged according to the means and SE (Fig. 1). Since the trapping tests were conducted within different times or blocks. The difference of population level in times or blocks may cause error to the result if the data were used directly. We used the trapping data of 0.5 mg and 1 mg sex pheromone dispensers (the dispensers were renewed each week) as to adjust our original trapping data to eliminate the error induced by the difference of the population level. Those loading rates were proved to be the effective monitoring tools to indicate the population level properly. In 2013, the experiment was conducted within same orchard at different times with a completely randomized design. The population variance between the various times required correction. The corrected data for each trap were the original trapping data/(the trapping data of the 0.5 mg + 1 mg dispensers). The 0.5 mg dispenser was not set for the overwinter generation. Therefore, its data were corrected as follows: original trapping data/(1 mg dispenser data × 2). The trapping data from the 1 mg dispenser were used to compensate for the missing value for the 0.5 mg dispenser. In fact, in the other experiment, there was no significant difference between the trapping data for the 0.5 mg and 1 mg dispensers, indicating the reliability of the correction method (Appendix Table 4). In 2014, the experiments were conducted in 3 orchards at various times, using a randomized block design. It was necessary to correct the population variance between the different times and blocks. The corrected data for each trap were the original trapping data/(the trapping data of the 0.5 mg + 1 mg dispensers within the same block).

The corrected trapping data were arranged according to the mean for a general additive model (gam) and a pruned exact linear time (PELT)^8^ analysis (Appendix Table 4) (because some of the corrected data were 0.05 or less than 0.05 adults/traps/day, all of the data were rounded to 2 decimal places). All analysis was carried out in the R environment (Version: 3.2.2). Both the corrected trapping data and the release rate data were log(*x*) transformed to normalize the variance (whereas only the corrected trapping data in the 2013 overwinter generation experiment were sqrt(*x*) transformed due to the occurrence of zero captures). First, a gam fit was used to analyze the main trend of the relationship between codling moth capture and sex pheromone release rate. The R^2^, deviance explanation, F value, P value and AIC value were obtained. Once the trend was determined, a PELT analysis was applied to search the sex pheromone release rates points that could cause significant change in catches (increase or decrease) and to divide the release rates into different intervals according to catches. The pheromone release rate that resulted in the maximum catches (V_0_) was defined as the optimal sex pheromone release rate. V_0_ could catch significantly more adults than lower (V_1_) or higher (V_2_) release rates. The V_0_ for each generation and the general V_0_ were obtained by simply determining the intersection of the V_0_ for the different years or for the different generations.

## Results

### Trapping test of different sex pheromone release rates in 2013

**Figure 2:**
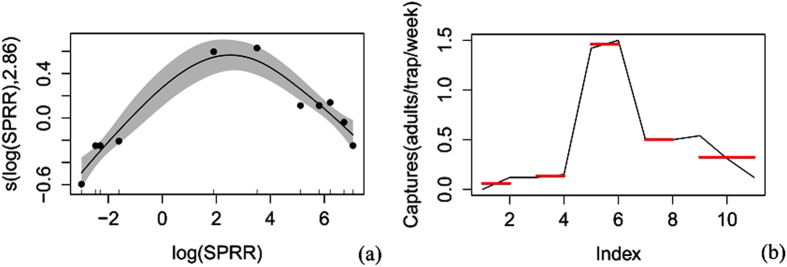
The analysis of the capture of males by different sex pheromone release rates in the 2013 overwinter generation. (**a**) Trend analysis of the generalized additive model; SPRR = sex pheromone release rate; the shaded area indicates the 95% confidence bands; (**b**) The pruned exact linear time analysis; Index = the order of the sex pheromone release rates from low to high; red lines indicate significant differences in moth capture.

V_0_ for the overwinter generation. The results for the overwinter generation in 2013 suggested that the male catches first increased and then decreased along with the increase in sex pheromone release rate (Fig. 2 and Table 1). When the sex pheromone release rates ranged from 0.050 to 1169.0 μg wk^−1^, 4 change points (0.084 μg wk^−1^, 0.2 μg wk^−1^, 33.4 μg wk^−1^ and 334.0 μg wk^−1^) were determined for the male catches (Method: PELT, SIC Value = 4.796). The release rates were divided into 5 segments: 0.050–0.084 μg wk^−1^, 0.1–0.2 μg wk^−1^, 6.7–33.4 μg wk^−1^, 167.0–334.0 μg wk^−1^, and 501.0–1169.0 μg wk^−1^. V_0_ was 6.7–33.4 μg wk^−1^, and its corresponding male catches were 1.46 ± 0.22 adults/trap/week. V_1_ and V_2_ were 0.050–0.2 μg wk^−1^ and 167.0–1169.0 μg wk^−1^, respectively. Their male catches were 0.10 ± 0.04 adults/week/trap and 0.39 ± 0.07 adult/week/trap, respectively, which were 6.8% and 26.7% of the catches for V_0_. The catches for V_0_ were 14.6 and 3.7 times those of V_1_ and V_2_, respectively.

**Figure 3:**
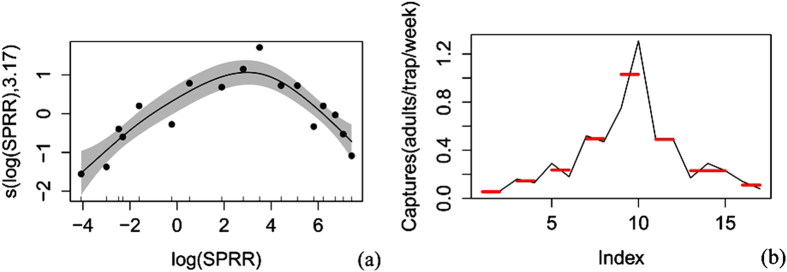
The analysis of the capture of males by different sex pheromone release rates in the 2013 first generation (**a**) Trend analysis of the generalized additive model; SPRR = sex pheromone release rates; the shaded area indicates the 95% confidence bands; (**b**) The pruned exact linear time analysis; Index = the order of the sex pheromone release rates from low to high; red lines indicate a significant difference in moth capture.

V_0_ for the first generation. The results for the first generation in 2013 indicated that the male catches first increased and then decreased along with the increase in sex pheromone release rate (Fig. 3 and Table 1). When the release rates ranged from 0.017 to 1670.0 μg wk^−1^, 7 change points (0.050 μg wk^−1^, 0.1 μg wk^−1^, 0.8 μg wk^−1^, 6.7 μg wk^−1^, 33.4 μg wk^−1^, 167.0 μg wk^−1^, and 835.0 μg wk^−1^) were detected for the male catches (PELT, SIC Value = 5.666). The release rates were divided into 8 segments: 0.017–0.050 μg wk^−1^, 0.084–0.1 μg/wk^−1^, 0.2–0.8 μg wk^−1^, 1.7–6.7 μg wk^−1^, 16.7–33.4 μg wk^−1^, 83.5–167.0 μg wk^−1^, 334.0–835.0 μg wk^−1^ and 1169.0–1670.0 μg wk^−1^. V_0_ was 16.7–33.4 μg week^−1^, and its corresponding male catches were 1.03 ± 0.16 adults/trap/week. V_1_ and V_2_ were 0.017–6.7 μg week^−1^ and 83.5–1670 μg week^−1^, respectively. Their male catches were 0.23 ± 0.05 adults/week/trap and 0.27 ± 0.04 adults/week/trap, respectively, which were 22.3% and 26.2% of the catches of V_0_. The catches for V_0_ were 4.5 and 3.8 times those of V_1_ and V_2_, respectively.

The general V_0_ for 2013 was obtained by simply determining the intersection of the V_0_ for the overwinter generation and for the first generation. For the sex pheromone release rates of 0.017–1670.0 μg wk^−1^, the general V_0_ for 2013 was 16.7–33.4 μg wk^−1^, and its corresponding male catches were 1.19 ± 0.16 adults/week/trap. V_1_ and V_2_ were 0.017–6.7 μg wk^−1^ and 83.5–1670.0 μg wk^−1^, respectively. Their catches were 0.28 ± 0.06 adults/week/trap and 0.32 ± 0.04 adults/week/trap, respectively, which were 23.5% and 26.9% of the catches for V_0_. The catches for V_0_ were 4.3 and 3.7 times those of V_1_ and V_2_, respectively.

### Trapping test of different sex pheromone release rates in 2014

**Figure 4:**
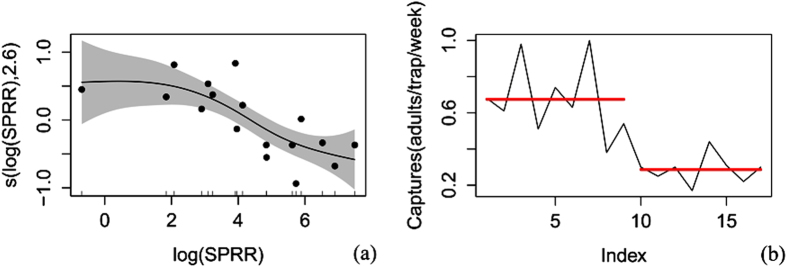
The analysis of the capture of males by different sex pheromone release rates in the 2014 overwinter generation (**a**) Trend analysis of the generalized additive model; SPRR = sex pheromone release rate; the shaded area indicates the 95% confidence bands; (**b**) The pruned exact linear time analysis; Index = the order of the sex pheromone release rates from low to high; red lines indicate a significant difference in moth capture.

V_0_ for the overwinter generation. The results for the overwinter generation in 2014 suggested that the male catches first remained constant and then decreased along with the increase in sex pheromone release rate (Fig. 4 and Table 1). When the release rates ranged from 0.5 to 1797.9 μg wk^−1^, only one change point (62.3 μg wk^−1^) was detected (PELT, SIC Value = 5.666). The release rates were divided into 2 segments: 0.5–62.3 μg wk^−1^ and 126.5–1797.9 μg wk^−1^. V_0_ was 0.5–62.3 μg wk^−1^, and its corresponding male catches were 0.67 ± 0.06 adults/trap/week. V_2_ was 126.5–1797.9 μg wk^−1^ and its corresponding male catches were 0.29 ± 0.03 adults/week/trap. The catches for V_2_ were 43.3% of the catches for V_0_; the catches for V_0_ were 2.3 times those of V_2_.

**Figure 5:**
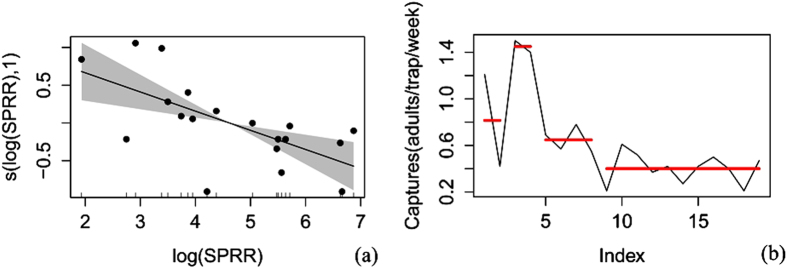
The analysis of the capture of males by different sex pheromone release rates in the 2013 first generation (**a**) Trend analysis of the generalized additive model; SPRR = sex pheromone release rate; the shaded area indicates the 95% confidence bands; (**b**) The pruned exact linear time analysis; Index = the order of the sex pheromone release rates from low to high; red lines indicate a significant difference in moth capture.

V_0_ for the first generation. The results of the first generation in 2014 indicated that male catches decreased gradually along with the increase in sex pheromone release rate (Fig. 5 and Table 1). When release rates ranged from 6.9 to 965.3 μg wk^−1^, 3 change points (15.6 μg wk^−1^, 29.6 μg wk^−1^, and 51.9 μg wk^−1^) were detected (PELT, SIC Value = 5.889). The release rates were divided into 4 segments: 6.9–15.6 μg wk^−1^, 18.4–29.6 μg wk^−1^, 33.1–51.9 μg wk^−1^, and 67.4–965.3 μg wk^−1^. V_0_ was 18.4–29.6 μg wk^−1^, and its corresponding male catches were 1.45 ± 0.29 adults/trap/week. V_1_ and V_2_ were 6.9–15.6 μg wk^−1^ and 33.1–965.3 μg wk^−1^, respectively. The male catches of V_1_ and V_2_ were 0.82 ± 0.19 adults/trap/week and 0.49 ± 0.04 adults/trap/week, respectively, which were 56.6% and 33.8% of the catches for V_0_. The captures for V_0_ were 1.8 and 3.0 times those of V_1_ and V_2_, respectively.

There were few differences between the results of the gam and PELT analyses. The gam analysis did not detect the increase in male catches when the release rates ranged from 6.9–15.6 μg wk^−1^ to 18.4–29.6 μg wk^−1^. This result could have occurred because the unreasonably low moth catches of 15.6 μg wk^−1^ may have had a negative effect on the gam fitting. Therefore, the PELT result was chosen to more reasonably determine V_0_.

The general V_0_ for 2014 was obtained by simply determining the intersection of V_0_ for the overwinter generation and first generation. When the sex pheromone release rates ranged from 0.5 to 1797.9 μg wk^−1^, V_0_ was 16.7–33.4 μg wk^−1^ and its corresponding male catches were 1.19 ± 0.16 adults/trap/week. V_1_ and V_2_ were 0.5–6.3 μg wk^−1^ and 51.9–1797.9 μg wk^−1^, respectively, and their corresponding catches were 0.73 ± 0.09 adults/trap/week and 0.43 ± 0.03 adults/trap/week, which were 68.2% and 40.2% of the catches for V_0_. The captures for V_0_ were 1.6 and 2.8 times those of V_1_ and V_2_, respectively.

### General V_0_ for 2013 + 2014

Overwinter generation. The general V_0_ for the overwinter generation was obtained by simply determining the intersection of the V_0_ for the 2013 and 2014 overwinter generations. When the sex pheromone release rates ranged from 0.050 to 1797.9 μg wk^−1^, the V_0_ for the overwinter generation was 6.7–33.4 μg wk^−1^, and its corresponding catches were 0.82 ± 0.11 adults/trap/week. V_1_ and V_2_ were 0.050–6.3 μg wk^−1^ and 50.1–1797.9 μg wk^−1^, respectively. Their captures were 0.43 ± 0.07 adults/trap/week and 0.38 ± 0.03 adults/trap/week, respectively, which were 52.4% and 46.3% of the V_0_. The captures for V_0_ were 1.9 and 2.2 times those of V_1_ and V_2_, respectively.

First generation. The general V_0_ for the first generation was obtained by simply determining the intersection of V_0_ for the 2013 and 2014 first generations. When the sex pheromone release rates ranged from 0.017 to 1670.0 μg wk^−1^, the V_0_ for the overwinter generation was 18.4–29.6 μg wk^−1^, and its corresponding catches were 1.45 ± 0.29 adults/trap/week. V_1_ and V_2_ were 0.017–16.7 μg wk^−1^ and 33.1–1670.0 μg wk^−1^, respectively. Their captures were 0.50 ± 0.09 adults/trap/week and 0.46 ± 0.03 adults/trap/week, respectively, which were 34.5% and 31.7% of the captures for V_0_. The captures for V_0_ were 2.9 and 3.2 times those of V_1_ and V_2_, respectively.

Final V_0_ for codling moth. The final V_0_ for the codling moth was obtained by simply determining the intersection of V_0_ for the overwinter generation and the first generation. For the sex pheromone release rate range of 0.017–1797.9 μg wk^−1^, V_0_ was 18.4–29.6 μg wk^−1^ and its corresponding male catches were 1.07 ± 0.06 adults/trap/week. V_1_ and V_2_ were 0.017–18.2 μg wk^−1^ and 33.1–1797.9 μg wk^−1^, respectively. Their captures were 0.55 ± 0.06 adults/trap/week and 0.44 ± 0.02 adults/trap/week, respectively, which were 51.4% and 41.1% those of the V_0_. The captures for V_0_ were 1.9 and 2.4 times those of V_1_ and V_2_, respectively.

All of the results obtained from each gam + PELT analysis suggested that regardless of the generation or year analyzed, the V_0_ was similar. However, there was some variance. All of the V_0_ ranged from 0.5–62.3 μg wk^−1^. The narrowest range of values was obtained for the first generation of 2014, for which the V_0_ was 18.4–29.6 μg wk^−1^. The widest range of values was obtained for the overwinter generation in 2014, for which the V_0_ was 0.5–62.3 μg wk^−1^. To ensure the accuracy of the results, the intersection of V_0_ over years and generations was determined step by step, and the final V_0_ for the codling moth was defined as 18.4–29.6 μg wk^−1^ (Table 2).

The V_0_ range for the first generation was generally narrower than that for the overwinter generation. In 2013, the V_0_ for the overwinter generation was 6.7–33.4 μg wk^−1^ and the V_0_ for the first generation was 16.7–33.4 μg wk^−1^; in 2014, the V_0_ for the overwinter generation was 0.5–62.3 μg wk^−1^ and the V_0_ for the first generation was 18.4–29.6 μg wk^−1^; for the combination of 2013 + 2014, the V_0_ for the overwinter generation was 6.7–33.4 μg wk^−1^ and the V_0_ for the first generation was 18.4–29.6 μg wk^−1^ (Table 2).

As a proportion of V_0_, the catches for V_1_ or V_2_ differed in each experiment. The lowest was 6.8% for the 2013 overwinter generation, and the highest was 68.2% for the 2014 general analysis. Currently, the lack of quantitative criteria for distinguishing V_0_ from V_1_ and V_2_ commonly causes difficulties in pheromone release rate studies. Therefore, we determined whether the release rates were optimal according to the following criterion: if the male catches for a given release rate were less than 70% of the V_0_ obtained using 68.2% experiment data, then that release rate was not optimal (Table 2).

## Discussion

### The method chosen for testing the release rate in the experiment

The sex pheromone release rates were calculated by subtracting the sex pheromone amount after trapping from the sex pheromone amount before trapping. It could represent what actually happened in the field. Although the real sex pheromone release rate for dispenser might decrease as the pheromone amount reduced. In a short time, the release rate fells very little. For example, the 0.2 mg dispenser might release about 33.4 μg wk^−1^ in its first week. If we continue to use it in the field it might release about 27.8 μg wk^−1^. It only decreased 5.6 μg wk^−1^. That was the decline brought by 1 week. If we take instantaneous release in to consideration, like days or hours, the decline might be slight. It could deem that the pheromone release rate from dispenser was relatively constant in a week.

Currently, examining the residual amount of extraction of the dispenser was the optimal method to determine the actual sex pheromone release rate. This method takes 1 week interval as an appropriate time. If we take day or hour as time intervals it might not detect the difference between initial amount and residual amount because the release rate was so small in a day or an hour. For example 8.0 μg wk^−1^ could be detected, but 1.1 μg d^−1^ and 0.05 μg h^−1^ might not be detected and caused a lot of error. Another method was to use a push and pull system to collect the headspace sex pheromone. Since that method was set in enclosed space, the tested release rates were far less than the real release rates in the field. The method likes estimation by equation was discard the environmental effects in the whole grown season and the release rates still might be different from the actual release rate in the field. Solid phase micro extraction (SPME) is a good way for pheromone analysis. But it need stable temperature, extraction time and space to ensure the accuracy. Our research was carried out in the field and the environment conditions always changed make that method to be unsuitable.

### The differences in the design of our experiment between 2013 and 2014

The traps were set in 2013 and 2014 using a different experimental design. In 2013, the traps were set using a completely randomized design, and all traps were randomly set in one orchard. In 2014, the traps were set using a randomized block design; each orchard was divided into 5 blocks, and the traps were randomly set in each block. The reason for the different design was to adapt to the different experimental conditions of these two years. In 2013, the experiment was only conducted in 1 orchard; the trees were evenly distributed in this orchard, so the variance of the field environment was relatively small. Therefore, the completely randomized design was reasonable. In 2014, the experiment was carried out in 3 orchards, and the trees in each orchard were relatively unevenly distributed. Therefore, a randomized block design was more suitable to avoid environmental interference. In addition to the reasons mentioned above, the experiment in 2014 required much more field work. The randomized block design was convenient for trap setting and data recording, which made the work highly efficient.

### The optimal pheromone release rate may vary based on different sex pheromone purity, geographic populations or host races

The optimal sex pheromone release rate was 18.4–29.6 μg wk^−1^ in our study. In a previous study^7^, it was 77–469 μg wk^−1^. The difference between the two studies might result from the following 3 reasons.

First, the purity of the sex pheromone used in previous study was different from our research. We used >97% sex pheromone and they used 93% sex pheromone to prepare the dispenser. 4% in difference means Vacas *et al.* (2013) used relatively lower purity of sex pheromone. Their sex pheromone might have isomers or additional components. But they didn’t mention that whether they have other components or not. Our sex pheromone only contain E, E-8, 10-dodecadien-1-ol according to the test. Therefore we suppose that might partially explain the difference.

Second, the codling moth is a native pest in Spain but an invasive pest in China. In our field experiment location, the codling moth was first reported in 2006 and has colonized that location for nearly 10 years. In Lepidopteran pests, geographic variation in communication in moths has been widely reported, e.g., for *Heliothis subflexa* and *Heliothis virescens*^9^, *Ostrinia furnacalis*^10^ and *Spodoptera frugiperda*^11^. These variations were usually caused by the various proportions of different components of the sex pheromones between the geographic populations. The sex pheromone of the codling moth has 1 major component but several minor components, such as dodecanol and tetradecanol^1^,^12^. Currently, the sex pheromone has been found in different geographic populations of the codling moth; differences exist not only in the total amount of the female sex pheromone but also in the proportion of dodecanol^13^. For example, the females of Italy population and Netherland population produce lower amount sex pheromone than Canada and Spain population. Therefore we suppose that the corresponding males might have different sensibility for sex pheromone. If females produced relatively low amount of sex pheromone, the males might have higher sensibility. So when we use artificial sex pheromone, the optimum sex pheromone release rate for trapping will be different too.

Third, the cultivars of the orchard of the previous study^7^ differed from the cultivars of our study. The cultured varieties in their study were e.g., Royal Gala and Golden Suprema, whereas ours were K9 apple, 123 apple, and 5DN pear. Difference in host race with sex pheromone amount or components’ proportion has not been reported in codling moth. But it has been reported in other Lepidoptera pest such as *Spodoptera frugiperda*^14^, corn-strain female produce higher amount of sex pheromone component like Z7-12:OAC and Z9-12:OAC than rice-strain. We suppose that the similar phenomenon might exist in codling moth as well. That could also explain the difference just like we interpret with geographical population.

The geographic populations or host races were the key factors that most difficult to be proved in the field and also the key factors need to be understood for the sex pheromone release technique.

### The competition between sex pheromone released by artificial dispensers and that released by adult females

A sex pheromone dispenser can capture males because the artificially released sex pheromone can compete with the sex pheromone released by female adults. The dispenser can release sex pheromone for 24 h consecutively, but its release efficiency may be affected by wind, temperature and rainfall. In contrast, the adult females can adjust their sex pheromone release behavior based on the environment. In general, the codling moth can release approximately 6.5 ng h^−1^ over 2 hours of calling^15^. Based on the assumption that the female moth can release sex pheromone every day, its maximum sex pheromone release rate would be 6.5 ng h^−1^ × 2 h × 7 d ≈ 0.09 μg wk^−1^. In our study, the V_0_ was 6.7–33.4 μg wk^−1^ for the overwinter generation, which is 74.4–371.1 times that of female release. The V_0_ was 18.4–29.6 μg wk^−1^ for the first generation, which is 204.4–328.9 times that of female release. The final V_0_ was 18.4–29.6 μg wk^−1^, suggesting that when the artificial sex pheromone dispenser reached approximately 200–300 times the release rates of the females, it could completely outcompetes the female moths. It has been reported that the *lobesia borana* female could release about 0.3 ng h^−1^ sex pheromone^16^ and we assume that it could release about 0.3 ng h^−1^ × 2 h × 7 d ≈ 0.0042 μg wk^−1^. The optimum release rate for *lobesia borana* in the field had been reported as 2800 μg wk^−1^(400 μg d^−1^)^17^. Those results suggested that the artificial sex pheromone released from dispenser should reach approximately 6.6 × 10^5^ times of the female’s could outcompete female moth. The type of the sex pheromone of *Cydia pomonella* and *Lobesia borana* are different. The former belongs to the alcohols and the latter belongs to the esters. The difference of the physicochemical property might be the cause of the difference. But to fully understand it, more researches need to be done. Releasing artificial sex pheromone to outcompete the female as far as we could gives great benefits. For monitoring, we could get the pest population information that closer to the real condition. For controlling, we could trap the male adults as much as possible to reduce the females’ mating opportunity. It will control the pest in the certain extent.

### The difference in the optimal pheromone release rates between the different codling moth generations

In all analyses, the range of the optimal sex pheromone release rate for the overwinter generation was wider than for the first generation. That observation might be explained in 3 ways. First, the overwinter generation occurred in spring, and the first generation occurred in summer; the higher temperatures during the summer might cause the rapid dissipation of sex pheromones in the air; thus, in the overwinter generation in 2014, the low release rates, such as 0.5 μg wk^−1^, might not have produced the same trapping ability as in the first generation. Second, in summer, the sensitivity of the moth to physiological status might have been greater than in spring. The higher release rates, such as 62.3 μg wk^−1^, may not have been as attractive to the overwinter generation in 2014 as to the first generation. Because of the moth’s increased sensitivity to physiological status in warmer environments, the response to the larger sex pheromone release rates could shift from attraction to rejection. The smaller sex pheromone release rates might be easily affected by the insects’ physiological status, and larger sex pheromone release rates might be easily affected by environmental conditions. Third, the overwinter generation of the codling moth emerged over a short time; the age and physiological status of the adults were relatively consistent, so the optimum sex pheromone release rates might span a wider range.

A future monitoring program should consider the overwinter generation and the first generation differently. For example, dispensers made of same materials that have different sex pheromone release rates could be provided for the two generations. According to previous studies, the sex pheromone release rate of the rubber dispenser is proportional to the loading rate and is inversely proportional to the hang time^4^,^6^. Thus, for the overwinter generation, a rubber dispenser with a higher loading rate and low replacement frequency, which can provide a wider range of sex pheromone release rates, is suitable. For example, we could the dispenser which loading rate was 0.2 mg and renewed the dispenser every two weeks. It could release about 27.8–33.4 μg wk^−1^ sex pheromone in two weeks according to the estimation and the trapping ability remained optimal. For the first generation, a rubber dispenser with a lower loading rate and high replacement frequency, which can provide a narrower range of sex pheromone release rates, can be used. For example, we applied the dispenser with loading rate of 0.12 mg and renewed the dispenser once a week. It could release about 20.0 μg wk^−1^ sex pheromone in a week. Therefore we could achieve labor saving in overwinter generation by decrease the dispensers’ replacement frequency. Moreover, the overwinter generation of the codling moth occurs over a short period of time, so the dispenser should be efficient for 1 month. The first generation of the codling moth occurs over a long time, so a dispenser with a longer efficiency time can be achieved by increasing the loading rate of sex pheromone.

### The minimum sex pheromone release rate to disrupt mating

Mating disruption is widely applied for codling moth control^1^. Large sex pheromone release rates might cause the competitive attraction and sensory overload, leading to mating disruption^18^,^19^. The sex pheromone release rates that effectively disrupt mating should not have any male catches. In our study, the maximum sex pheromone release rate used was 1797.9 μg wk^−1^, and its corresponding catches were 2.0 ± 0.4 adults/trap (15 traps) in a week; this release rate still failed to reach the minimum sex pheromone release rate for mating disruption. A previous study^7^ used a maximum sex pheromone release rate of 7546 μg wk^−1^ and captured fewer than 0.1 adults/trap. To date, the minimum sex pheromone release rate for mating disruption remain unknown. Currently, the general release rate of codlemone for mating disruption is 0.6–41.6 μg h^−1^ (100.8–6988.8 μg wk^−1^)^20^,^21^, and this release rate was achieved using large numbers of dispensers, approximately 1000–2000 dispensers/ha^7^.

## Additional Information

**How to cite this article**: Liu, W. *et al.* The optimal sex pheromone release rate for trapping the codling moth *Cydia pomonella* (Lepidoptera: Tortricidae) in the field. *Sci. Rep.* **6**, 21081; doi: 10.1038/srep21081 (2016).

## Supplementary Material

Appendix tables

## Figures and Tables

**Table 1:** The generalized additive model used to fit the different sex pheromone release rates with their corresponding captures.

Experiment	R^2^	DE (%)	F	P	AIC
2013og	0.909	93.5	27.38	<0.001	−9.980629
2013fg	0.810	84.7	16.84	<0.001	21.98217
2014og	0.556	62.8	6.73	<0.01	19.48487
2014fg	0.394	42.8	12.70	<0.01	27.78024

^a^og = overwinter generation, fg = first generation, DE = deviance explanation.

**Table 2:** The V_0_ for codling moth for different generations and years.

Time/Generation	SPRR (μg/wk^−1^)	V_0_ (capture) (μg wk^−1^) (adults/trap/week)	V_1_(μg wk^−1^)	V_2_(μg wk^−1^)	Proportion (%)
2013og	0.050–1169.0*	6.7–33.4 (1.46)	0.050–0.2	167.0–1169.0	6.8/26.7
2013fg	0.017–1670.0*	16.7–33.4 (1.03)	0.017–6.7	83.5–1670	22.3/26.2
2013og + fg	0.017–1670.0*	16.7–33.4 (1.19)	0.017–1.7	83.5–1670.0	23.5/26.9
2014og	0.5–1797.9	0.5–62.3 (0.67)	—	126.5–1797.9	—/43.3
2014fg	6.9–965.3	18.4–29.6 (1.45)	6.9–15.6	33.1–965.3	56.6/33.8
2014og + fg	0.5–1797.9	18.4–29.6 (1.07)	0.5–6.3	51.9–1797.9	68.2/40.2
og2013 + 2014	0.050–1797.9**	6.7–33.4 (0.82)	0.050–6.3	52.4–1797.9	52.4/46.3
fg2013 + 2014	0.017–1670.0**	18.4–29.6 (1.45)	0.017–6.7	42.2–1670.0	34.5/31.7
(og + fg)(2013 + 2014)	0.017–1797.9**	18.4–29.6 (1.07)	0.017–6.3	51.9–1797.9	51.4/41.1

^a^*Sex pheromone release rates were estimated, **sex pheromone release rates were either estimated or actually tested.

^b^og = overwinter generation, fg = first generation, SPRR = sex pheromone release rate, Proportion = the proportion of V_0_ capture relative to V_1_ or V_2_; — means none.

## References

[b1] Witzgall P., Stelinski L., Gut L. & Thomson D. Codling moth management and chemical ecology. Annu. Rev. Entomol. 53, 503–522 (2008). 17877451 10.1146/annurev.ento.53.103106.093323

[b2] Knight A. L. In Area Wide Pest Management: Theory and Implementation (eds Koul O., Cuperus G. W. & Elliott N.) 159–190 (Wallingford, 2008).

[b3] Zhang R. *et al.* Progress on monitoring and control of the codling moth, *Cydia pomonella* (L.). *Chin*. J. Appl. Entomol. 49, 37–42 (2012).

[b4] Kehat M., Anshelevich L., Dunkelblum E., Fraishtat P. & Greeberg S. Sex pheromone traps for monitoring the codling moth: effect of dispenser type, field aging of dispenser, pheromone dose and type of trap on male captures. Entomol. Exp. Appl. 70, 55–62 (1994).

[b5] Mitchell V. J., Manning L. A., Cole L., Suckling D. M. & El-sayed A. M. Efficacy of the pear ester as a monitoring tool for codling moth *Cydia pomonella* (Lepidoptera: Tortricidae) in New Zealand apple orchards. *Pest* Manage. Sci. 64, 209–214 (2008). 10.1002/ps.1479 18189264

[b6] Zhu H. Y., Du L., Xu J., Liu W. & Zhang R. Effective duration of sex pheromone in delta traps for monitoring the codling moth. Chin. J. Appl. Entomol. 49, 114–120 (2012).

[b7] Vacas S., Miñarro M., Bosch M. D., Primo J. & Navarro-Llopis V. Studies on the codling moth (Lepidoptera: Torticidae) response to different codlemone release rates. Environ. Entomol. 42, 1383–1389 (2013). 24280412 10.1603/EN13114

[b8] Killick R. & Eckley I. A. Change point: An R package for change point analysis. J. Stat. Solftw. 58, 1–19 (2014).

[b9] Groot A. T. *et al.* Geographic and temporal variation in moth chemical communication. Evolution 63, 1987–2003 (2009). 19473383 10.1111/j.1558-5646.2009.00702.x

[b10] Huang Y. P. *et al.* Geographic variation in sex pheromone of Asian corn borer, *Ostrinia furnacalis*, in Japan. J. Chem. Ecol. 24, 2079–2088 (1998).

[b11] Unbehend M. *et al.* Geographic variation in sexual attraction of *Spodoptera frugiperda* Corn- and Rice-strain males to pheromone lures. PlosOne 9, 1–11 (2014). 10.1371/journal.pone.0089255 PMC3929749 24586634

[b12] Witzgall P. *et al.* Identification of further sex pheromone synergists in the codling moth, Cydia pomonella. Entomol. Exp. Appl. 101, 131–141 (2001).

[b13] Duménil C. *et al.* Intraspecific variation in female sex pheromone of the codling moth *Cydia pomonella*. Insects 5, 705–721 (2014). 26462935 10.3390/insects5040705 PMC4592601

[b14] Unbehend M., Hänniger S., Meagher R. L., Heckel D. G. & Groot A. T. Pheromonal divergence between two strains of *Spodoptera frugiperda*. J. Chem. Ecol. 39, 364–376 (2013). 23456344 10.1007/s10886-013-0263-6

[b15] Bäckman A. C., Bengtsson M. & Witzgall P. Pheromone release by individual females of codling moth, Cydia pomonella. J. Chem. Ecol. 23, 807–815 (1997).

[b16] Anfora G. *et al.* Attractiveness of year-old polyethylene Isonet sex pheromone dispensers for *Lobesia botrana*. Entomol. Exp. Appl. 117, 201–207 (2005).

[b17] Vacas S., Alfaro C., Zarzo M., Navarro-Llopis V. & Primo J. Effect of sex pheromone emission on the attraction of *Lobesia botrana*. Entomol. Exp. Appl. 139, 250–257 (2011).

[b18] Cardé R. T. & Minks A. K. Control of moth pests by mating disruption, successes and constraints. Annu. Rev. Entomol. 40, 559–585 (1995).

[b19] Miller J. R. *et al.* General principles of attraction and competitive as revealed by large-cage studies of moths responding to sex pheromone. PNAS 107, 22–27 (2010). 20018720 10.1073/pnas.0908453107 PMC2806766

[b20] Brown D. F. *et al.* Emission characteristics of a polyethylene pheromone dispenser for mating disruption of codling moth (Lepidoptera: Tortricidae). J. Econ. Entomol. 85, 910–917 (1992).

[b21] Knight A. L. Evaluating pheromone emission rate and blend in disrupting sexual communication of codling moth (Lepidoptera: Tortricidae). Environ. Entomol. 24(6), 1396–1403 (1995).

