# Supplementary material for: The optimal sex pheromone release rate for trapping the codling moth Cydia pomonella (Lepidoptera: Tortricidae) in the field
Source: Sci Rep. 2016 Feb 16;6:21081. doi: 10.1038/srep21081 (PMC4754646; doi:10.1038/srep21081)
Supplement: Appendix tables [file srep21081-s1.doc]

Appendix tables 1-4

Title: The optimal sex pheromone release rate for trapping the codling moth *Cydia pomonella* (Lepidoptera: Tortricidae) in the field

**Wei Liu, ^a, b^ Jing Xu, ^a^ Runzhi Zhang, ^a, c*^**

^a^ CAS Key Laboratory of Zoological Systematics and Evolution, Institute of Zoology, Chinese Academy of Sciences, #1 Beichen West Rd., Chaoyang, Beijing 100101, China.

^b^ University of Chinese Academy of Sciences, Beijing 100049, P. R. China

^c^ State Key Laboratory of Integrated Management of Pest Insects and Rodents, Beijing 100101, China

* Corresponding author. Email: [zhangrz@ioz.ac.cn](mailto:zhangrz@ioz.ac.cn)

Appendix table 1. Estimation of the dispensers’ sex pheromone release rates in 2013

| Loading rate (mg) | Sprr (μg wk^-1^) | Loading rate (mg) | Sprr (μg wk-^1^) |
| --- | --- | --- | --- |
| 0.0001 | 0.017 | 0.2* | 33.4 |
| 0.0003* | 0.050 | 0.5 | 83.5 |
| 0.0005* | 0.084 | 1* | 167.0 |
| 0.0008* | 0.1 | 2* | 334.0 |
| 0.001* | 0.2 | 3* | 501.0 |
| 0.005 | 0.8 | 5* | 835.0 |
| 0.01 | 1.7 | 5+2* | 1169.0 |
| 0.04* | 6.7 | 5+5 | 1670.0 |
| 0.1 | 16.7 |  |  |

^a^ *Sex pheromone loading rates set only for the overwinter generation; the other sex pheromone loading rates were set for both the overwinter and first generations.

^b^ Sprr = Sex pheromone release rate.

Appendix table 2. Actual testing of the sex pheromone release rates for the combinations of different dispenser loading rates and pre-hang times in 2014

| Generation | # | Combination (loading rates×pre-hanged times), sex pheromone amount (μg), sex pheromone release rates (μg wk^-1^) | | | | | | | | | | |
| --- | --- | --- | --- | --- | --- | --- | --- | --- | --- | --- | --- | --- |
| Overwinter generation | 1 | Combination | 0.05 (0) | 0.1 (0) | 0.2 (0) | 0.5 (0) | 1 (0) | 2 (0) | 3 (0) | 5 (0) | 10 (0) | / |
|  |  | Ia | 66.9±1.3 | 122.1±1.4 | 181.3±2.5* | 447.7±26.4 | 792.4±12.0 | 1818.9±11.5** | 3026.1±18.2 | 4540.6±18.8 | 8458.4±70.9 | / |
|  |  | Ra | 58.9±0.7 | 96.7±2.4 | 159.2±2.5 | 397.6±10.7* | 730.1±19.3 | 1692.4±11.5** | 2717.7±45.2 | 4180.3±60.4 | 7776.9±118.6 | / |
|  |  | Sprr | 8.0 | 25.4 | 22.1 | 50.1 | 62.3 | 126.5 | 308.4 | 360.3 | 681.5 | / |
|  | 2 | Combination | 0.05 (4) | 0.1 (4) | 0.2 (4) | 0.5 (4) | 1 (4) | 2 (4) | 5 (4) | 10 (4) | / | / |
|  |  | Ia | 51.6±1.4** | 86.5±3.8 | 155.1±3.3 | 414.7±15.0 | 709.4±37.9* | 1675.3±23.0 | 3875.8±146.0 | 7349.8±75.3 | / | / |
|  |  | Ra | 51.1±0.4** | 80.2±1.6 | 136.9±4.0 | 362.3±20.5 | 581.8±15.2** | 1399.6±31.7 | 2888.2±263.7* | 5551.9±191.5 | / | / |
|  |  | Sprr | 0.5 | 6.3 | 18.2 | 52.4 | 127.6 | 275.7 | 987.6 | 1797.9 | / | / |
| First generation | 3 | Combination | 0.05 (0) | 0.1 (0) | 0.2 (0) | 0.5 (0) | 1 (0) | 2 (0) | 3 (0) | 5 (0) | 10 (0) | / |
|  |  | Ia | 46.3±0.9 | 98.5±2.3 | 172.3±2.5 | 435.1±12.9 | 901.3±17.0 | 1920.8±48.7 | 3177.1±32.5 | 3813.1±217.7 | 6946.7±189.9 | / |
|  |  | Ra | 39.4±1.0* | 80.1±2.0 | 142.7±3.2 | 355.3±4.5 | 747.5±17.5 | 1618.0±27.2 | 2394.8±53.6 | 3055.5±59.3** | 5981.4±84.8 | / |
|  |  | Sprr | 6.9 | 18.4 | 29.6 | 79.8 | 153.8 | 302.8 | 782.3 | 757.6 | 965.3 | / |
|  | 4 | Combination | 0.2 (0) | 0.2 (2) | 0.2 (4) | 0.2 (8) | 0.2 (12) | 1 (0) | 1 (2) | 1 (4) | 1 (8) | 1(12) |
|  |  | Ia | 191.7±2.7 | 232.3±15.9** | 180.8±3.4 | 173.3±8.2 | 147.7±8.3 | 916.5±34.6 | 898.6±33.0 | 946.1±26.5 | 886.3±58.4 | 544.2±5.7 |
|  |  | Ra | 144.0±1.4 | 180.4±8.6* | 147.7±5.4 | 131.1±6.1 | 132.1±6.3 | 671.6±21.2 | 659.7±14.6 | 665.6±26.7 | 625.6±25.5 | 476.8±19.4 |
|  |  | Sprr | 47.7 | 51.9 | 33.1 | 42.2 | 15.6 | 244.9 | 238.9 | 280.5 | 260.7 | 67.4 |

^a^ Ia=Initial amount of sex pheromone in the dispenser before trapping, Ra=Residual amount of sex pheromone in the dispenser after trapping, Sprr=Sex pheromone release rate

^b^ Im and Rm are reported as the mean±SE (*n=4; **n=3; for others, n=5)

^c^ Sprr=Ia-Ra

Appendix table 3. The efficiency of sex pheromone extraction from the dispenser

| Sample | Ea (μg) | Ta (μg) | E (%) | Sample | Ea (μg) | Ta (μg) | E (%) |
| --- | --- | --- | --- | --- | --- | --- | --- |
| y-0.1-j-1 | 61.5 | 116.4 | 52.8 | y-10-c-2 | 3879.5 | 7552.3 | 51.4 |
| y-0.1-c-4 | 47.2 | 93.3 | 50.6 | y-0.1(4)-j-1 | 44.0 | 89.3 | 49.3 |
| y-0.2-j-1 | 91.5 | 184.8 | 49.5 | y-0.1(4)-c-1 | 38.2 | 75.3 | 50.7 |
| y-0.2-c-2 | 82.4 | 159.8 | 51.6 | y-0.2(4)-j-3 | 78.2 | 157.3 | 49.7 |
| y-0.5-j-3 | 214.6 | 411.4 | 52.2 | y-0.2(4)-c-1 | 67.6 | 135.9 | 49.7 |
| y-0.5-c-2 | 203.6 | 389.6 | 52.3 | y-0.5(4)-j-3 | 203.8 | 403.9 | 50.5 |
| y-1-j-3 | 379.6 | 750.5 | 50.6 | y-0.5(4)-c-1 | 176.8 | 363.0 | 48.7 |
| y-1-c-2 | 362.8 | 691.3 | 52.5 | y-1(4)-j-3 | 359.5 | 754.9 | 47.6 |
| y-2-j-1 | 905.6 | 1914.4 | 47.3 | y-1(4)-c-3 | 291.1 | 571.5 | 50.9 |
| y-2-c-1 | 852.7 | 1645.1 | 51.9 | y-2(4)-j-3 | 800.9 | 1716.4 | 46.7 |
| y-3-j-1 | 1535.9 | 3042.3 | 50.5 | y-2(4)-c-1 | 658.4 | 1399.2 | 47.1 |
| y-3-c-4 | 1415.2 | 2675.7 | 52.9 | y-3(4)-j-5 | 1460.8 | 2915.0 | 50.1 |
| y-5-j-2 | 2312.6 | 4412.5 | 52.4 | y-3(4)-c-3 | 1125.8 | 2339.4 | 48.1 |
| y-5-c-2 | 2184.7 | 4085.1 | 53.5 | y-5(4)-j-5 | 2086.0 | 4101.5 | 50.9 |
| y-10-j-5 | 4331.9 | 8631.0 | 50.2 | y-5(4)-c-1 | 1475.6 | 2895.2 | 51.9 |

^a^ Ea=Extracted amount of sex pheromone, Ta=Total amount of sex pheromone, E=Efficiency

^b^ The average efficiency of sex pheromone extraction for the dispenser was 50.4±1.8% (n=30).

^c^ The total amount of sex pheromone was calculated as the sum of the sex pheromone amounts by 5 times extraction. Each extraction method was the same as mentioned in 2.4.2.1(The 0.05-3 mg sex pheromone dispensers were extracted in 2 ml vials with 1 ml hexane for 24 h. The 5 mg and 10 mg sex pheromone dispensers were extracted in 4 ml vials with 3 ml hexane for 24 h). After 5 times extraction, almost no sex pheromone remained in the extraction solvent.

Appendix table 4.1. The corrected capture data for the different sex pheromone release rates in 2013

| Time/Generation | Data (Sprr: μg wk^-1^, Capture: adults/trap/week) | | | | | | | | | | | | | | |
| --- | --- | --- | --- | --- | --- | --- | --- | --- | --- | --- | --- | --- | --- | --- | --- |
| 2013og | Sprr | 0.050 | 0.084 | 0.1 | 0.2 | 6.7 | 33.4 | 167.0 | 334.0 | 501.0 | 835.0 | 1169.0 |  |  |  |
|  | Capture | 0.00 | 0.12 | 0.12 | 0.15 | 1.42 | 1.50 | 0.50 | 0.50 | 0.54 | 0.31 | 0.12 |  |  |  |
| 2013fg | Sprr | 0.017 | 0.050 | 0.084 | 0.1 | 0.2 | 0.8 | 1.7 | 6.7 | 16.7 | 33.4 | 83.5 | 167.0 | 334.0 | 501.0 |
|  | Capture | 0.05 | 0.06 | 0.16 | 0.13 | 0.29 | 0.18 | 0.52 | 0.47 | 0.75 | 1.31 | 0.49 | 0.49 | 0.17 | 0.29 |
|  | Sprr | 835.0 | 1169.0 | 1670.0 |  |  |  |  |  |  |  |  |  |  |  |
|  | Capture | 0.23 | 0.14 | 0.08 |  |  |  |  |  |  |  |  |  |  |  |
| 2013og+fg | Sprr | 0.017 | 0.050 | 0.084 | 0.1 | 0.2 | 0.8 | 1.7 | 6.7 | 16.7 | 33.4 | 83.5 | 167.0 | 334.0 | 501.0 |
|  | Capture | 0.05 | 0.03 | 0.14 | 0.12 | 0.22 | 0.18 | 0.52 | 0.95 | 0.75 | 1.41 | 0.49 | 0.50 | 0.34 | 0.42 |
|  | Sprr | 835.0 | 1169.0 | 1670.0 |  |  |  |  |  |  |  |  |  |  |  |
|  | Capture | 0.27 | 0.13 | 0.08 |  |  |  |  |  |  |  |  |  |  |  |

^a^ Sprr=Sex pheromone release rate, og=overwinter generation, fg=first generation

^b^ The corrected capture data are reported as the mean (2013og, 2013fg, n=5; 2013og+fg, sex pheromone release rates set for both the overwinter generation and the first generation, n=10; sex pheromone release rates set only for the first generation, n=5)

^c^ Sex pheromone release rates in 2013 were estimated; therefore, dispensers with the same loading rates had the same pheromone release rates for the 2 generations.

Appendix table 4.2. The corrected capture data for different sex pheromone release rates in 2014

| Time/Generation | Data (Sprr: μg wk^-1^, Capture: adults/trap/week) | | | | | | | | | | | | | | |
| --- | --- | --- | --- | --- | --- | --- | --- | --- | --- | --- | --- | --- | --- | --- | --- |
| 2014og | Sprr | 0.5 | 6.3 | 8.0 | 18.2 | 22.1 | 25.4 | 50.1 | 52.4 | 62.3 | 126.5 | 127.6 | 275.7 | 308.4 | 360.3 |
|  | Capture | 0.68 | 0.61 | 0.98 | 0.51 | 0.74 | 0.63 | 1.00 | 0.38 | 0.54 | 0.30 | 0.25 | 0.30 | 0.17 | 0.44 |
|  | Sprr | 681.5 | 987.6 | 1797.9 |  |  |  |  |  |  |  |  |  |  |  |
|  | Capture | 0.31 | 0.22 | 0.30 |  |  |  |  |  |  |  |  |  |  |  |
| 2014fg | Sprr | 6.9 | 15.6 | 18.4 | 29.6 | 33.1 | 42.2 | 47.7 | 51.9 | 67.4 | 79.8 | 153.8 | 238.9 | 244.9 | 260.7 |
|  | Capture | 1.21 | 0.42 | 1.50 | 1.40 | 0.69 | 0.57 | 0.78 | 0.55 | 0.21 | 0.61 | 0.52 | 0.37 | 0.42 | 0.27 |
|  | Sprr | 280.5 | 302.8 | 757.6 | 782.3 | 965.3 |  |  |  |  |  |  |  |  |  |
|  | Capture | 0.42 | 0.50 | 0.40 | 0.21 | 0.47 |  |  |  |  |  |  |  |  |  |
| 2014og+fg | Sprr | 0.5 | 6.3 | 6.9 | 8.0 | 15.6 | 18.2 | 18.4 | 22.1 | 25.4 | 29.6 | 33.1 | 42.2 | 47.7 | 50.1 |
|  | Capture | 0.68 | 0.61 | 1.21 | 0.98 | 0.42 | 0.51 | 1.50 | 0.74 | 0.63 | 1.40 | 0.69 | 0.57 | 0.78 | 1.00 |
|  | Sprr | 51.9 | 52.4 | 62.3 | 67.4 | 79.8 | 126.5 | 127.6 | 153.8 | 238.9 | 244.9 | 260.7 | 275.7 | 280.5 | 302.8 |
|  | Capture | 0.55 | 0.38 | 0.54 | 0.21 | 0.61 | 0.30 | 0.25 | 0.52 | 0.37 | 0.42 | 0.27 | 0.30 | 0.42 | 0.50 |
|  | Sprr | 308.4 | 360.3 | 681.5 | 757.6 | 782.3 | 965.3 | 987.6 | 1797.9 |  |  |  |  |  |  |
|  | Capture | 0.17 | 0.44 | 0.31 | 0.40 | 0.21 | 0.47 | 0.22 | 0.30 |  |  |  |  |  |  |

^a^ Sprr= Sex pheromone release rates, og= overwinter generation, fg= first generation

^b^ The corrected capture data was mean (n=15)

Appendix table 4.3. The corrected capture data for different sex pheromone release rates for 2013+2014

| Time/Generation | Data (Sprr: μg wk^-1^, Capture: adults/trap/week) | | | | | | | | | | | | | | |
| --- | --- | --- | --- | --- | --- | --- | --- | --- | --- | --- | --- | --- | --- | --- | --- |
| 2013+2014  og | Sprr | 0.050 | 0.084 | 0.1 | 0.2 | 0.5 | 6.3 | 6.7 | 8.0 | 18.2 | 22.1 | 25.4 | 33.4 | 50.1 | 52.4 |
|  | Capture | 0.00 | 0.12 | 0.12 | 0.15 | 0.68 | 0.61 | 1.42 | 0.98 | 0.51 | 0.74 | 0.63 | 1.50 | 1.00 | 0.38 |
|  | Sprr | 62.3 | 126.5 | 127.6 | 167.0 | 275.7 | 308.4 | 334.0 | 360.3 | 501.0 | 681.5 | 835.0 | 987.6 | 1169.0 | 1797.9 |
|  | Capture | 0.54 | 0.30 | 0.25 | 0.50 | 0.30 | 0.17 | 0.50 | 0.44 | 0.54 | 0.31 | 0.31 | 0.22 | 0.12 | 0.30 |
| 2013+2014  fg | Sprr | 0.017 | 0.050 | 0.084 | 0.1 | 0.2 | 0.8 | 1.7 | 6.7 | 6.9 | 15.6 | 16.7 | 18.4 | 29.6 | 33.1 |
|  | Capture | 0.05 | 0.06 | 0.16 | 0.13 | 0.29 | 0.18 | 0.52 | 0.47 | 1.21 | 0.42 | 0.75 | 1.50 | 1.40 | 0.69 |
|  | Sprr | 33.4 | 42.2 | 47.7 | 51.9 | 67.4 | 79.8 | 83.5 | 153.8 | 167.0 | 238.9 | 244.9 | 260.7 | 280.5 | 302.8 |
|  | Capture | 1.31 | 0.57 | 0.78 | 0.55 | 0.21 | 0.61 | 0.49 | 0.52 | 0.49 | 0.37 | 0.42 | 0.27 | 0.42 | 0.50 |
|  | Sprr | 334.0 | 501.0 | 757.6 | 782.3 | 835.0 | 965.3 | 1169.0 | 1670.0 |  |  |  |  |  |  |
|  | Capture | 0.17 | 0.29 | 0.40 | 0.21 | 0.23 | 0.47 | 0.14 | 0.08 |  |  |  |  |  |  |
| 2013+2014  og+fg | Sprr | 0.017 | 0.050 | 0.084 | 0.1 | 0.2 | 0.5 | 0.8 | 1.7 | 6.3 | 6.7 | 6.9 | 8.0 | 15.6 | 16.7 |
|  | Capture | 0.05 | 0.03 | 0.14 | 0.12 | 0.22 | 0.68 | 0.18 | 0.52 | 0.61 | 0.95 | 1.21 | 0.98 | 0.42 | 0.75 |
|  | Sprr | 18.2 | 18.4 | 22.1 | 25.4 | 29.6 | 33.1 | 33.4 | 42.2 | 47.7 | 50.1 | 51.9 | 52.4 | 62.3 | 67.4 |
|  | Capture | 0.51 | 1.50 | 0.74 | 0.63 | 1.40 | 0.69 | 1.41 | 0.57 | 0.78 | 1.00 | 0.55 | 0.38 | 0.54 | 0.21 |
|  | Sprr | 79.8 | 83.5 | 126.5 | 127.6 | 153.8 | 167.0 | 238.9 | 244.9 | 260.7 | 275.7 | 280.5 | 302.8 | 308.4 | 334.0 |
|  | Capture | 0.61 | 0.49 | 0.30 | 0.25 | 0.52 | 0.50 | 0.37 | 0.42 | 0.27 | 0.30 | 0.42 | 0.50 | 0.17 | 0.34 |
|  | Sprr | 360.3 | 501.0 | 681.5 | 757.6 | 782.3 | 835.0 | 965.3 | 987.6 | 1169.0 | 1670.0 | 1797.9 |  |  |  |
|  | Capture | 0.44 | 0.42 | 0.31 | 0.40 | 0.21 | 0.27 | 0.47 | 0.22 | 0.13 | 0.08 | 0.30 |  |  |  |

^a^ Sprr= Sex pheromone release rates, og= overwinter generation, fg= first generation

^b^ The corrected capture data was mean (2013+2014og: 2013og, n=5, 2014og, n=15; 2013+2014fg: 2013fg, n=5, 2014fg, n=15; 2013+2014 og+fg: 2013og+fg, the release rates set both in overwinter generation and first generation, n=10, the release rates only set in overwinter generation, n=5, 2014og+fg, n=15)
